# Ecological Predictors and Trajectory of Internet Addiction from Childhood through Adolescence: A Nationally Representative Longitudinal Study

**DOI:** 10.3390/ijerph18126253

**Published:** 2021-06-09

**Authors:** Yi-Ping Hsieh, Hsiao-Lin Hwa, April Chiung-Tao Shen, Hsi-Sheng Wei, Jui-Ying Feng, Ching-Yu Huang

**Affiliations:** 1 Department of Social Work, University of North Dakota, Grand Forks, ND 58202, USA; 2 Department and Graduate Institute of Forensic Medicine, National Taiwan University, Taipei 100, Taiwan; hwahl013@ntu.edu.tw; 3 Department of Social Work, National Taiwan University, Taipei 106, Taiwan; acshen@ntu.edu.tw (A.C.-T.S.); hswei@mail.ntpu.edu.tw (H.-S.W.); 4 Department of Nursing, College of Medicine, National Cheng Kung University and Hospital, Tainan City 701, Taiwan; juiying@mail.ncku.edu.tw; 5 School of Psychology, Keele University, Staffordshire ST5 5BG, UK; soarhuang@gmail.com

**Keywords:** internet addiction, resilience, neglect, school experience, community violence

## Abstract

We examined multidimensional factors within four systems (individual, family, school, and community) that influence internet addiction across time among children through adolescence in Taiwan. We hypothesize that internet addiction increases from childhood to adolescence and that resilience, child neglect, positive school experiences, and community violence are significant predictors at baseline and of the rate of change across time. Based on stratified random sampling, a valid sample size of 6233 Taiwanese children participated in our study, which we began in 2014 and then followed this sample in 2016 and 2018 using repeated measures. We used hierarchical linear modeling to model changes in internet addiction across time (with equal two-year intervals between assessments) and the associations between the predictors and internet addiction over time. The results show that internet addiction increases from childhood to adolescence. After controlling for gender, we found that resilience and positive school experiences predict less internet addiction, whereas neglect and community violence predict greater internet addiction. Over time, greater resilience predicts a decreasing trajectory of internet addiction, whereas greater neglect and community violence predict a slower increasing trajectory and positive school experiences predict a faster-increasing trajectory. A holistic approach can help children cope with internet addiction.

## 1. Introduction

### 1.1. Internet Addiction

Internet addiction refers to the inability to control internet use and can eventually lead to psychological, social, and vocational problems and impairment [1,2]. The core symptoms of internet addiction include compulsive symptoms, withdrawal symptoms, and tolerance of internet addiction [3]. Researchers have associated internet addiction with sleep problems [4], psychiatric disorders, mental health problems [5,6], aggressive behaviors [7], problematic alcohol use [8], and even suicidality [9]. Meta-analysis results from a 2014 study indicate that the overall prevalence of internet addiction among samples from 31 nations in seven world regions (ages 12–41, mean = 18.42 years) is 6%, with 10.9% in the Middle East, 8% in North America, and 7.1% in Asia [10]. Although the prevalence of internet addiction varies across cultures and societies and might be measured differently using various instruments, Young et al. [11] found that the overall prevalence is 4.6% to 4.7% among adolescents and 13% to 18.4% among college students. In Taiwan, the prevalence of internet addiction is 15.8% to 18.7% among adolescents [9,12,13]. In sum, internet addiction is common among adolescents worldwide and can have negative impacts on their physical, psychological, and behavioral development.

The dynamic systems model of developmental psychopathology [14] emphasizes the importance of understanding ways that maladaptive psychological functioning/behaviors (e.g., internet addiction) emerge and change over time in a coactive person-environment system. Huang [15] found stability rather than a change in internet addiction among college students, but other studies have shown a change in problematic internet use across time among adolescents [16] and college students [17]. Although most studies of internet addiction have focused on adults, college students, and adolescents, less is known about internet addiction among school-age children and changes in internet addiction over time from childhood through adolescence. The transition from childhood to adolescence is marked by various developmental issues and has been referred to as an ‘age of storm and stress’ [18]. Early adolescence is a developmental period of rapid change in terms of physical, psychological, social, and cognitive aspects and is the transitional time when identity and autonomy are established [19]. To address the increased stress that is present during this transitional period, adolescents may escape and try to cope by diving into the cyber world, and they may become addicted to the internet [20]. Thus, we hypothesize that internet addiction has an increasing trajectory across time from childhood to adolescence.

### 1.2. Ecological Factors and Internet Addiction

Bronfenbrenner’s [21] ecological systems theory emphasizes the interrelationship among different processes and their contextual variation and has been widely adopted for theoretical frameworks. From microsystems, mesosystems, and exosystems to macrosystems, these systems are connected by social interactions that link contexts within systems and across systems (e.g., individual, family, school, community, and time). Bronfenbrenner [22] argued that meaning and behaviors are created or exhibited by the developing person as a product of all their experiences. The measurement of social context is essential to understanding the development of individuals. Thus, to understand the risk and protective factors that are associated with internet addiction, we need to consider ecological factors in the contexts of individual, family, school, community, and time. Here, we aim to examine the roles of individual resilience, child neglect, positive school experiences, and community violence as predictors of internet addiction across time from childhood through adolescence.

Resilience is a complex concept with various definitions and is considered a protective factor of internet addiction [23]. Many of the definitions of ‘resilience’ share common features, such as human strength, adaptive coping, and positive outcomes following exposure to adversity [24,25,26]. Resilience refers to the ability to bounce back from negative emotional experiences and adapt with flexibility to the changing demands of stressful experiences [27]. For example, Hjemdal et al. [28] related higher levels of resilience to lower levels of depression, anxiety, stress, and obsessive-compulsive symptoms. Hsieh et al. [29] related adverse childhood experiences, such as neglect and physical and sexual violence, to psychological distress (e.g., depression and anxiety) and, in turn, to internet addiction among children. In short, as a protective factor that reduces the likelihood of internet addiction, resilience can be considered a buffer against the negative effects of stressful experiences on internet addiction. That is, resilience reduces psychological distress and thus serves to break the pathway from the stress caused by adverse childhood experiences to internet addiction.

Child neglect, on the other hand, is considered a risk factor for internet addiction [29] and smartphone addiction [30]. Child neglect is the most common type of child maltreatment [31] and is defined as “failure to provide for the development of the child in all spheres: health, education, emotional development, nutrition, shelter, and safe living conditions… and causes or has a high probability of causing harm to the child’s health or physical, mental, spiritual, moral or social development” [32]. The Longitudinal Studies of Child Abuse and Neglect (LONGSCAN) research team included failure to provide necessities, such as food, clothing, shelter, and medical care, and lack of supervision in its definition of child neglect [33]. Stoltenborgh et al. [34] reported that the prevalence of child physical neglect is 16.3% and emotional neglect is 18.4 percent. Neglected children demonstrate lower peer interaction than physically abused children [35], and physical neglect is associated with internalizing symptomatology and withdrawn behavior [36]. In addition to the negative impacts on social interaction and psychological wellbeing, child neglect also has direct and indirect effects on internet addiction through psychological symptoms [20]. When children are neglected, they exhibit higher levels of psychological symptoms and in turn, are more likely to become addicted to the internet to temporarily reduce the stress and seek comfort [20].

In addition to the protective factor of resilience, a positive school experience also can function as a protective buffer against internet addiction. Social control theory [37] suggests that ties or bonds to family, school, or community diminish and inhibit social deviance. When children’s or adolescents’ bonds to school are strong and school experiences are positive, these positive school experiences might be able to prevent or limit the likelihood of internet addiction. Similarly, stage-environment fit theory [38] suggests that adolescents whose social environments and contexts adequately respond to and satisfy their developmental needs are more likely to experience positive outcomes. Adolescents whose positive school experiences respond to their developmental needs could also limit the likelihood of internet addiction. In contrast, adolescents who decline school engagement tend to engage in increased delinquency and substance use over time [39]. In addition to substance use as a negative outcome, Tas [40] found a negative correlation between school engagement and internet addiction. As school engagement levels decrease, internet addiction increases. Adolescents who feel connected to school or perceive the school climate as favorable are less likely to develop problematic internet use [41] and problematic online game use [42].

Lastly, community violence is considered another risk factor for internet addiction. Community violence refers to violence that occurs in the child’s environment but outside the home and between or among individuals who are not members of the child’s family [43]. Community violence exposure includes witnessing or experiencing violent behavior (e.g., a physical threat, robbery, possession of a weapon, shooting, or stabbing) [44,45]. Adolescents who are exposed to community violence may exhibit psychological and behavioral problems [46,47,48]. For example, Sullivan et al. [49] have found that witnessing violence predicts subsequent substance use initiation among rural adolescents, and the experience or witness of community violence may increase the likelihood of using substances to cope with the aftermath of the event. General strain theory [50] suggests that experiences of strain or stress generate negative emotions and in turn create pressure for corrective action, such as maladaptive coping behaviors, to escape from the source of the adversity. Community violence exposure has been linked to various addictive behaviors and internet gaming disorders among adolescents [51,52]. Thus, community violence exposure may increase adolescents’ strain and negative emotions (e.g., fear, anxiety, anger), which in turn may lead them to develop internet addiction as a coping strategy to escape adversity in the real world.

### 1.3. Study Aims

**Figure 1:**
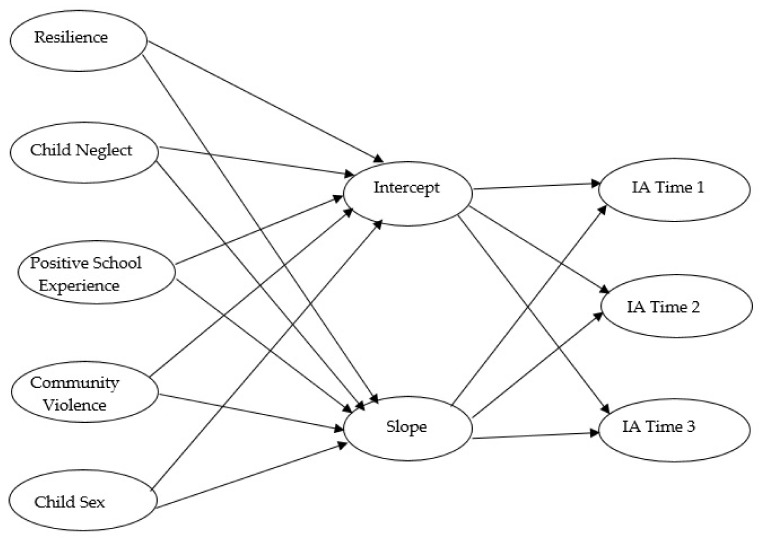
Hypothesized Research Model. *Note.* IA = internet addiction. Time 1 and Time 2 are two years apart. Time 2 and Time 3 are 2 years apart.

Based on ecological system theory, we examined multidimensional factors through which four interconnected systems, namely, individual, family, school, and community, influence the likelihood of internet addiction among children through adolescence in Taiwan. Specifically, we examined whether the trajectory of internet addiction increases across time from childhood to adolescence and whether resilience, child neglect, a positive school experience, and community violence predict internet addiction at baseline and the rate of change across time. We hypothesize that the trajectory of internet addiction will rise across time from childhood to adolescence. We also hypothesize that higher levels of resilience and a positive school experience will predict lower levels of internet addiction at baseline and a slower rate of change, whereas child neglect and community violence will predict higher levels of internet addiction at baseline and a faster rate of change across time. Figure 1 illustrates the research model.

## 2. Methods

### 2.1. Participants

We conducted this study in the spring semesters of 2014, 2016, and 2018. We followed 6233 fourth-graders (10 to 11 years old) over five years through eighth grade. Before collecting the formal data, we conducted a pilot study in the fall semester of 2013 with 726 fourth-graders to test the psychometrics of the measures. We then modified some survey items according to the students’ feedback and the reliability and validity analysis results obtained from the pilot study. To enhance the formal sample representations, we used a stratified random sampling method. We stratified the formal sample proportionately by county and city in Taiwan (a total of 19 counties and cities). We first divided these counties and cities into urban and rural areas and then randomly selected districts within each area. Via contact with school principals, we invited children from all the primary schools in each selected district to participate in the study. Half of the invited schools agreed to participate in this study. We noted geographical differences between the participating schools and non-participating schools. The average school participation rates in northern Taiwan were lower than the average national school participation rates. The major reasons given for not participating in the study included that the school focused on accelerated academic performance and disapproved of any activities that may detract from their students’ learning time. Using proportionately stratified random sampling, we contacted 314 elementary schools whose principals agreed to participate in the study, and 99.9% of the children in those schools (*n* = 6290) agreed to participate, with parental consent. After excluding 57 subjects for incomplete signatures of either parent/guardian or children, we had a final total of 6233 Taiwanese children who participated in the longitudinal study in 2014 (Time 1). Two years later in 2016, when these participants became sixth-graders (Time 2), the valid sample size was 3737. Another two years later in 2018, when the participants became eighth-graders (Time 3), the valid sample size was 2661. We used baseline (Time 1), two-year follow-up (Time 2), and four-year follow-up (Time 3) data in the present study.

### 2.2. Procedures

Prior to sampling and data collection, we obtained approval for this study from the Research Ethics Committee of the National Taiwan University Hospital. Research assistants then contacted the principals of the participating schools for consent. Students were asked to take home an introduction letter and a consent form that requested their parents’ signatures and to return the signed form to their teachers. We also obtained informed consent forms from all participating students. We distributed self-report questionnaires to consenting students in group sessions at the schools during Times 1, 2, and 3 (which were two years apart). The questionnaires in three waves were anonymous without any personal information on them. We assigned an ID code for each participant at baseline. The questionnaires in each wave were collected along with the consent forms (with contact information and signature). Only the staff in the research center has the access to the consent form. After placing a sticker with a matching ID code on each questionnaire, the consent forms were separated from the questionnaires to ensure anonymity and were kept in a secure place by the center staff. The center staff followed students in three waves by the contact information provided in the consent form. After obtaining parental consent, center staff provided the name list of consenting students for teachers to arrange for a group survey. When this group of students turned 14 years old (became eighth-graders in Time 3), they transitioned to middle schools. We contacted their new schools and went through the same procedure, with a few exceptions that included completing the survey outside the school upon parents’ requests. Students received stationery as a gift for their participation. 

### 2.3. Measures

We assessed children’s resilience, neglect, positive school experiences, perceived community violence, and internet addiction using self-reported survey responses during the three-time points across five years. A group of seven multidisciplinary experts (including child development scholars, a sociologist, a clinical social worker, and a statistician) examined the questionnaire for content validity. We then administered the validated questionnaire to 726 students in a pilot study. The research team modified some of the questions based on internal consistency analysis and principal component analysis results obtained from the pilot study as well as suggestions from scholars and other experts. The research team examined the psychometrics of these measures again after collecting the formal data. The findings support those of previous studies that school-age children (especially ages 8 to 12) can reliably report their own health status, violence-victimization experience, and quality of life [53,54,55].

#### 2.3.1. Resilience

We used the 10-item Connor-Davidson Resilience Scale (CD-RISC) [56] to measure an individual’s ability to thrive despite adversity. Students rated items on a rating scale from 1 (never like that) to 5 (always like that). We reverse-coded two items and computed mean scores; higher scores indicate greater resilience. The scale demonstrated strong internal consistency (α = 0.80) in this study.

#### 2.3.2. Child Neglect

We used four modified items from the International Society for the Prevention of Child Abuse & Neglect (*ISPCAN)* Child Abuse Screening Tool Children’s Version (ICAST-C) [57] to measure physical neglect, which includes having inadequate clothes, unmet medical needs, hunger, or thirst, and being left home alone, in the past year. Students rated the frequency of neglect by their parents on a 5-point scale (1 = never, 2 = 1–2 times, 3 = 3–5 times, 4 = 6–10 times, and 5 = more than 10 times). We recoded the response for each item to reflect the average number of times the neglect occurred. The recoded scale ranged from 0 to 10. We then computed mean scores; higher scores indicate higher levels of child neglect. The internal consistency of the scale (α = 0.61) is questionable but acceptable [58,59,60].

#### 2.3.3. Positive School Experience

We created six items as an ad-hoc questionnaire to measure positive school experience. Sample questions include: “I feel happy in school”, “I feel safe in school”, “I feel I am an accepted member of the school”, “Teachers do care about me”, and “Teachers in school are willing to listen to me when I try to say something.” Students rated the items on a scale from 1 (never feel this way) to 5 (always feel this way). We computed mean scores; higher scores indicate higher levels of the positive school experience. The scale demonstrated strong internal consistency (α = 0.87) in this study.

#### 2.3.4. Perceived Community Violence

We used two items modified from the Child Community Violence Scale items of the University of California at Los Angeles Post Traumatic Stress Disorder Index for Diagnostic and Statistical Manual of Mental Disorders-IV (UCLA PTSD Index for DSM-IV) [61] to measure children’s perceptions of community violence in the past year, including being beaten or threatened to be hurt badly in the community or witnessing someone being beaten or threatened to be hurt badly in the community. Students rated the frequency of community violence on a scale from 1 (never) to 5 (more than 10 times). We then recoded the scores into dummy variables (0 = no such experience and 1 = have such experience).

#### 2.3.5. Internet Addiction

We used ten items adapted from the Chen Internet Addiction Scale (CIAS) [62] to measure internet addiction, which includes core systems and related problems. The core symptoms are compulsive symptoms, withdrawal symptoms, and tolerance, and the related problems are interpersonal/health problems and time-management problems. Students rated the items on a 5-point scale from 1 (very not true) to 5 (very true). We computed mean scores; higher scores indicate higher levels of internet addiction. The scale demonstrated strong internal consistency for all three time points, i.e., Times 1, 2, and 3 (α = 0.88, 0.89, 0.90, respectively).

### 2.4. Statistical Analyses

We used two-level multilevel modeling (hierarchical linear modeling) [63] to model changes across time [64] for internet addiction, with equal spacing between measurement occasions (3 data points in 6 years), and to model the associations between the independent variables and the child outcome (internet addiction) over time. We nested repeated measures of internet addiction within individuals. Level 1 of the model describes a child’s change over time (within-person), including the initial status (intercept) and the rate of change (slope). To provide a meaningful intercept and minimize collinearity between years [63], we centered time at Time 1 and coded Times 1, 2, and 3 as 0, 1, and 2, respectively. The first-year assessment thus represents the intercept. Level 2 describes the different patterns of change across children (between-person) based on the predictors.

First, we estimated unconditional growth models to examine whether significant variability in the trajectory of internet addiction was evident. We then modeled repeated assessments of internet addiction as a function of time. The variable ‘Time’ represents the time in years at each assessment and is within-child. In terms of variance, the Level 1 residual variance (σε2) summarizes the average scatter of a child’s observed outcome values around his or her own true change trajectory. The Level 2 variance components quantify the amount of unpredicted variation in the individual growth parameters; σ02 assesses the unpredicted variability in the true initial status and σ12 assesses the unpredicted variability in the true rate of change [64]. Second, we estimated conditional models to examine between-child associations between each of the four predictors (resilience, child neglect, positive school experiences, and community violence) and child outcome (internet addiction) across the elementary and middle school years when controlling for gender. To test whether between-child differences in both the intercept and the trajectory of internet addiction over time were associated with these four ecological levels of predictors, we added the four baseline measures as fixed effects in the model. In addition, we included gender in the models to estimate the between-child effects. We centered all the variables at Level 2 on the grand mean for the sample so that the Level 2 intercepts represented adjusted means for the average participants in the sample. Using grand mean centering gives the intercept and slope terms a meaningful interpretation. We tested these predictors simultaneously in the model so that each predictor brought its own unique contribution to the model.

We performed additional analyses to test whether attrition led to nonrandom sampling. That is, we tested whether participants who were staying in wave 3 differed from the participants who were missing. We then compared the two groups for each of the study variables. To assess attrition effects, we used multiple logistic regression to assess the presence of nonrandom sampling and independent sample *t*-tests to assess the effects of nonrandom sampling on the means [65]. If the results of the multiple logistic regression analysis indicated no nonrandom sampling, then data were missing at random, at least with respect to the variables of interest, and we could be reasonably confident that attrition would not bias our subsequent longitudinal data analysis of these variables [65]. If the results of the multiple logistic regression analysis indicated the presence of nonrandom sampling, then we would assess the effects of nonrandom sampling on the data using independent sample *t*-tests.

## 3. Results

The preliminary analysis results indicate that the study variables were not skewed (values between +2 and −2); however, child neglect in Time 1 was affected slightly by kurtosis (values slightly over 2) [66]. Although the distributions for child neglect show heavy tails, they are not considered problematic (value below 8) [67]. Assumptions of normality and linearity were not violated, and the predictors and dependent variables were not highly intercorrelated. Table 1 presents the means, standard deviations, and correlations for the study variables. Although resilience and positive school experiences are shown to be negatively correlated with internet addiction, child neglect is positively correlated with internet addiction in Times 1, 2, and 3. The prevalence rate of internet addiction in the sample at baseline is 13% based on the diagnostic cut-off point of the CIAS [68]. Results from an independent sample t test indicated that boys (M = 1.95, SD = 0.81) scored higher on Internet addiction at baseline than girls (M = 1.69, SD = 0.71), t(6111) = 13.06, *p* < 0.001, but no gender difference in Time 2 and Time 3. In addition, boys (M = 3.62, SD = 0.61) scored higher on resilience than girls (M = 3.49, SD = 0.60), t(2636) = 5.37, *p* < 0.001. Boys (M = 1.38, SD = 1.82) also scored higher on child neglect than girls (M = 1.06, SD = 1.50), t(6005) = 7.69, *p* < 0.001. Girls (M = 3.93, SD = 0.95) scored higher on positive school experience than boys (M = 3.82, SD = 1.01), t(6203) = −4.14, *p* < 0.001. In addition, children with community violence experience scored much higher on Internet addiction (M = 2.16, 2.02, 2.14, SD = 0.87, 0.82, 0.83) at baseline and Time 2 and Time 3 than children without such experience (M = 1.73, 1.81, 1.96, SD = 0.71, 0.72, 0.72), t(1881) = −16.73, t(1157) = −6.32, t(778) = −4.52 (*p* < 0.001), respectively. Children with community violence (M = 2.23, SD = 2.25) scored much higher on child neglect than children without such experience (M = 0.94, SD = 1.35), t(1621) = −20.06, *p* < 0.001. Lastly, children without community violence experience (M = 3.59, 3.96, SD = 0.59, 0.95) scored higher on resilience and positive school experience than children with such experience (M = 3.42, 3.58, SD = 0.65, 1.04), t(796) = 5.44, t(2004) = 11.98 (*p* < 0.001), respectively.

Overall, 57% of data were missing at time 3, so the attrition rate is 57 percent. Missing data were handled by the full information maximum likelihood procedure (FIML), which deals estimate parameters iteratively based on complete cases instead of imputing them [69]. To assess attrition effects, we used multiple logistic regression to assess the presence of nonrandom sampling and independent sample t-tests to assess the effects of nonrandom sampling on the means. The multiple logistic regression analysis results show no nonrandom sampling. Data are missing at random, at least with respect to the variables of interest, except for positive school experience. Thus, we are reasonably confident that attrition did not bias the longitudinal data analyses of our studied variables [65], except for positive school experience. To discern the effects of nonrandom sampling on the means, we performed independent sample t-tests. We compared the mean differences of positive school experience between participants who responded (stayers) versus those who did not respond (leavers) at Time 3. The results show that participants who were missing data and were not included in the analyses had lower levels of positive school experiences (M = 3.84, SD = 1) than those who stayed in the study in Time 3 (M = 3.93, SD = 0.95), t(5817) = −3.85, *p* < 0.001. However, with a large sample size and robust power, even a small difference between means may be statistically significant [65]. Although t-tests show a significant mean difference, a mean difference of 0.09 represents only 1.8% of the range of the 5-point Likert scale. Thus, we are reasonably confident that attrition did not bias the longitudinal data analyses of our studied variables.

### 3.1. Trajectory of Internet Addiction across Elementary and Middle School Years

We began this analysis by estimating unconditional growth models to determine whether significant variability was present at the initial status and that an increase in internet addiction was evident over time. The average internet addiction score at baseline for the average child in the sample is 1.77 (SE = 0.01, *p* < 0.001) on a 5-point rating scale. The average rate of change in internet addiction across the time points is 0.11 (SE = 0.01, *p* < 0.001). The positive and significant coefficient on the slope indicates that the trajectory of internet addiction increased across the elementary and middle school years (from the fourth grade to the eighth grade); the change per time point (two years apart) is 0.11. In terms of variance, the estimated within-person (Level 1) residual variance for internet addiction is 0.32 (*p* < 0.001), the estimated between-person (Level 2) residual variance for internet addiction in the true initial status is 0.25 (*p* < 0.001), and the estimated between-person residual variance in the true rate of change is 0.07 (*p* < 0.001). All of these variances are significant, indicating that internet addiction in the first year and the change in internet addiction across five years changed significantly across the sample.

### 3.2. Ecological Factors and Internet Addiction across Elementary and Middle School Years

We estimated a conditional model to examine between-person associations between each of the four ecological factors (resilience, child neglect, positive school experiences, and community violence) and the outcome of internet addiction (See Table 2). In the conditional model, the average internet addiction score at baseline for the average child in the sample is 1.84 (*p* < 0.001), and the average rate of change in internet addiction across time points is 0.08 (*p* < 0.001).

After controlling for gender, in terms of the individual-level factor, participants with greater resilience displayed lower levels of internet addiction at baseline (β = –0.12, *p* < 0.001) compared to other participants and showed a decreasing trajectory of internet addiction across time (β = –0.11, *p* < 0.001). In terms of the family-level factor, participants who perceived higher levels of child neglect reported higher levels of internet addiction at baseline (β = 0.07, *p* < 0.001) compared to other participants and showed a slower increasing trajectory of internet addiction across time (β = −0.03, *p* < 0.001). In terms of the school-level factor, participants who rated their school experience as more positive displayed lower levels of internet addiction at baseline (β = −0.14, *p* < 0.001) compared to other participants, but showed a faster-increasing trajectory of internet addiction across time (β = 0.04, *p* = 0.001). In terms of the community-level factor, participants who experienced or witnessed community violence reported higher levels of internet addiction at baseline (β = 0.12, *p* < 0.001) than other participants and showed a slower increasing trajectory of internet addiction across time (β = −0.05, *p* < 0.001). In terms of gender, boys experienced higher levels of internet addiction at baseline (β = 0.10, *p* < 0.001) than girls, but a slower increasing trajectory of internet addiction than girls across time (β = −0.04, *p* < 0.001).

## 4. Discussion

### 4.1. Primary Findings

In this study, we examined the trajectory of internet addiction across time from childhood to adolescence as well as the multidimensional factors by which four microcosmic systems, namely, individual, family, school, and community, influence the likelihood of internet addiction across time in a Taiwanese sample. The results indicate that internet addiction increases over time from childhood to adolescence. The results also support that higher levels of resilience and positive school experiences are predictors of less internet addiction, whereas higher levels of child neglect and community violence are predictors of greater internet addiction at baseline. Over time, greater resilience predicts a decreasing trajectory of internet addiction, whereas greater neglect and community violence predict a slower increasing trajectory and a positive school experience predicts a faster-increasing trajectory of internet addiction. As children grow into adolescence, we found that the level of internet addiction decreases for children with greater resilience and increases for children who report higher levels of neglect, community violence, and, unexpectedly, a positive school experience. We tried to determine the reasons for this unexpected finding. In the digital era, children and adolescents spend much time communicating with their peers via smartphones and online devices [70], and their internet activities are less likely to be restricted or regulated by their peers [71], which makes peers an important source of influence on adolescents’ internet-related attitudes and behaviors. Thus, a possible explanation for this unexpected result is that adolescents who reported positive school experiences have good relationships with their peers, are involved in peer interactions, and spend much time online with their peers. Such engagement may increase their vulnerability to online risks [72] and the likelihood of internet addiction as they transition from childhood to adolescence.

In line with ecological systems theory [21], four microcosmic systems (individual, family, school, and community) can negatively or positively affect the likelihood of internet addiction across time. First, consistent with a previous study [23], we identified individual resilience as a protective factor in predicting internet addiction. In the ‘age of storm and stress’, a higher level of resilience enhances adolescents’ ability to bounce back from negative emotional and stressful experiences and cope with stress and, in turn, reduces the likelihood of developing internet addiction. Second, in addition to the stress that children experience during the developmental transition to adolescence, the experience of child neglect accelerates their risk of psychological symptoms, which may lead adolescents to seek comfort and support in the cyber world and become addicted to the internet. Substitute internet-based social networks sometimes compensate for the social needs of children who are neglected in the real world. Children may find ways to cope in the cyber world to relieve real-life stress, reduce their psychological distress, and temporarily feel good about themselves. Consequently, these children may become increasingly reliant on internet use as a maladaptive coping strategy and become addicted to it. Third, in line with social control theory [37] and stage-environment fit theory [38], this study’s results suggest that positive school experiences adequately respond to and satisfy children’s and adolescents’ developmental needs and, in turn, diminish and inhibit the social deviance of internet addiction. Lastly, in line with general strain theory [50], our results suggest that the experience or witness of community violence generates negative emotions and creates pressure for corrective actions such as internet addiction to escape adversity and cope with the aftermath of the event. Internet addiction and substance dependence are both addictive behaviors and share several similar characteristics and risk factors. Similar to findings presented in substance abuse literature [73,74], the process of internet addiction also can be understood as a maladaptive coping strategy and a self-medicating behavior.

### 4.2. Strengths

The present study contributes to the literature regarding internet addiction by using an ecological systems theory model and a holistic approach to determine the predictors of internet addiction. Using a nationally representative longitudinal sample, we considered not only individual characteristics but also multidimensional social environments and contexts to understand the development of internet addiction across time from childhood to adolescence. The large sample size and stratified random sampling provided sufficient power for the analyses to test a relatively complete version of the ecological systems theory model and enhanced the sample representation. This study is the first to explore the roles of ecological factors in predicting the baseline and trajectory of internet addiction across developmental transitions in a Taiwanese sample. We examined the unique contributions and effects of four interconnected systems (individual, family, school, and community) on internet addiction using multilevel modeling with regard to both the initial status and the rate of change. By using multilevel modeling to model change(s) across time, we explored the intercept and slope in terms of internet addiction as well as the between-child differences and within-child differences as they changed over time. Moreover, having three assessments (in 2014, 2016, and 2018) over five successive years allowed us to follow fourth-graders through their eighth-grade year and to model changes in child outcomes across the developmental transition from childhood to adolescence.

### 4.3. Limitations

This study has several limitations. First, because children were the only source for reporting the predictors and outcome (internet addiction) using self-reported measures, the associations found between the predictors and outcome may be due to shared method variance. Second, the Cronbach’s alpha for child neglect is relatively low (α = 0.61); nonetheless, although this value is questionable, it is not poor or unacceptable [58,59,60]. The perceived community violence was measured using only two items adopted from the UCLA PTSD Index, and it can be a limitation for the measure validation. Nonetheless, Gardner et al. [75] have revealed that when comparing multiple-item, Likert-type measures of psychological constructs to single-item, non-Likert-type measures of the same constructs, neither method appeared to be empirically better than the other. Third, the attrition rate is 57% across the five years from fourth grade to eighth grade. Following children across time in a large, nationally representative random sample was challenging in this study, especially when the participants attended different schools for their elementary and middle school years, moved, changed phone numbers, studied abroad, etc. One reason for subject attrition is that some principals in previously participating schools or newly contacted middle schools declined to participate in the study in Time 2 or Time 3, so groups of participants/students in these schools were not followed and asked to participate in Time 2 and Time 3. Thus, the problem of subject attrition is more likely to be attributable to school administrative decisions than to the characteristics of the participants/students. Although subject attrition can be a possible source of bias that overestimates or underestimates relationships among study variables [76], in our study, we examined the effects of attrition and found no significant effect. Replication is needed in future studies to obtain greater validity to the findings which are more likely to be generalized to the larger population.

## 5. Conclusions

The results of our study provide empirical insights into the trends of internet addiction among children and adolescents across time in Taiwan and the effects of multidimensional factors on their internet addiction. Internet addiction often is complicated by the effects of ecological factors in the context. A holistic approach, which takes into account the whole interconnected system rather than just the behavior itself, can be helpful for children and adolescents to cope with internet addiction. Community education regarding the severity and consequences of internet addiction and its risk and protective factors is important to raise public awareness of these issues. Intervention programs can screen children for exposure to neglect and community violence and help foster children’s resilience to the negative effects of toxic stress and enhance positive school experiences, thus promoting well-being for children and adolescents in Taiwan and elsewhere.

## Figures and Tables

**Table 1:** Bivariate correlations, means, and standard deviations for study variables.

Study Variables	1.	2.	3.	4.	5.	6.
1. Resilience	--					
2. Child neglect	−0.14 ** ^1^	--				
3. Positive school experience	0.24 **	−0.20 **	--			
4. Internet addiction T1	−0.17 **	0.23 **	−0.22 **	--		
5. Internet addiction T2	−0.21 **	0.17 **	−0.19 **	0.32 **	--	
6. Internet addiction T3	−0.30 **	0.12 **	−0.17 **	0.20 **	0.43 **	--
Mean	3.56	1.22	3.88	1.82	1.86	1.20
SD ^2^	0.61	1.68	0.98	0.77	0.74	0.75
Scale range	1–5	0–10	1–5	1–5	1–5	1–5

^1^ ** *p* < 0.01. ^2^ SD = Standard Deviation.

**Table 2:** Multilevel models for change across time for internet addiction.

	Internet Addiction	
Study Variables	Coefficient	SE
Fixed Effects		
Initial status, π_0*i*_		
Intercept	1.84 ***	0.02
Gender	0.10 ***	0.01
Resilience	−0.12 ***	0.02
Child neglect	0.07 ***	0.01
Positive school experience	−0.14 ***	0.02
Community violence	0.12 ***	0.02
Rate of change, π_1*i*_		
Intercept	0.08 ***	0.01
Gender	−0.04 ***	0.01
Resilience	−0.11 ***	0.02
Child neglect	−0.03 ***	0.01
Positive school experience	0.04 ***	0.01
Community violence	−0.05 ***	0.01
Variance components		
Level 1		
Level 2		
Within-person, σ_ε_^2^	0.32 ***	
In initial status, σ_0_^2^	0.17 ***	
In rate of change, σ_1_^2^	0.06 ***	
Correlation between ζ_0*i*_ and ζ_1*i*_, τ	−0.48	

*** *p* < 0.001. Codes for gender are 1 = boys, −1 = girls. SE = Standard Error.

## Data Availability

The data presented in this study are available upon reasonable request due to restrictions. The data are not publicly available due to confidentiality.
